# BMI, HOMA-IR, and Fasting Blood Glucose Are Significant Predictors of Peripheral Nerve Dysfunction in Adult Overweight and Obese Nondiabetic Nepalese Individuals: A Study from Central Nepal

**DOI:** 10.1155/2016/2810158

**Published:** 2016-04-20

**Authors:** Lekhjung Thapa, P. V. S. Rana

**Affiliations:** ^1^National Institute of Neurological and Allied Sciences, Bansbari, Kathmandu 44606, Nepal; ^2^CMS-TH, Chitwan 44200, Nepal

## Abstract

*Objective.* Nondiabetic obese individuals have subclinical involvement of peripheral nerves. We report the factors predicting peripheral nerve function in overweight and obese nondiabetic Nepalese individuals.* Methodology.* In this cross-sectional study, we included 50 adult overweight and obese nondiabetic volunteers without features of peripheral neuropathy and 50 healthy volunteers to determine the normative nerve conduction data. In cases of abnormal function, the study population was classified on the basis of the number of nerves involved, namely, “<2” or “≥2.” Multivariable logistic regression analysis was carried out to predict outcomes.* Results.* Fasting blood glucose (FBG) was the significant predictor of motor nerve dysfunction (*P* = 0.039, 95% confidence interval (CI) = 1.003–1.127). Homeostatic model assessment of insulin resistance (HOMA-IR) was the significant predictor (*P* = 0.019, 96% CI = 1.420–49.322) of sensory nerve dysfunction. Body mass index (BMI) was the significant predictor (*P* = 0.034, 95% CI = 1.018–1.577) in case of ≥2 mixed nerves' involvement.* Conclusion.* FBG, HOMA-IR, and BMI were significant predictors of peripheral nerve dysfunction in overweight and obese Nepalese individuals.

## 1. Introduction

Peripheral neuropathy is a very common condition, which is often distressing and disabling [1]. The population prevalence of peripheral neuropathy is about 2.4%, rising with age to 8% [2]. Peripheral neuropathies are among the most common long-term complications of diabetes, affecting up to 50% [3]. However, peripheral neuropathy is linked not only to diabetes, but also to metabolic syndrome in these patients. Very close relation of obesity is found with insulin resistance and hyperinsulinemia [4]. In obese patients, various abnormalities of nerve functions are observed in routine electrophysiological testing. Asymptomatic neuropathies, decreased amplitude in NCS (nerve conduction studies), and subclinical involvement of different diameter sensory fibers have been established. These abnormalities are partly related to hyperinsulinemia and insulin sensitivity [5, 6]. We studied the predictors of abnormal peripheral nerve function in overweight and obese nondiabetic Nepalese individuals.

## 2. Materials and Methods

A cross-sectional study was conducted on 50 adult overweight or obese nondiabetic volunteers recruited from the outpatient department of College of Medical Sciences Teaching Hospital, Bharatpur, Nepal, after obtaining an informed consent. The ethical approval for this study was obtained from the institutional review board. Detail history and clinical examination findings of all the participants were recorded.

The data on height, weight, abdominal girth, and waist-hip ratio were obtained and the participants were classified into two groups using WHO (World Health Organization) criteria [7] of four groups: “obese group 1” (3 groups of WHO classification: “overweight,” “at risk,” and “obesity grade 1”) and “obese group 2” (4th group of WHO classification). The American Diabetic Association criteria were used to differentiate nondiabetics from diabetics [8]. Serum insulin was determined at fasting and 2 h after oral glucose tolerance test (OGTT). HOMA-IR was calculated using FBG and fasting insulin levels.

NCS was performed with Neuroperfect, 4-channel EMG/NCV/EP machine 2000 by Medicaid using surface electrode for motor nerve conduction and ring electrodes for sensory NCS in the air-conditioned neurophysiology lab with control of temperature to ideal and the details of the obtained values were systematically recorded.

Motor nerve conduction studies (MNCS) were done in median nerve (a supramaximal stimulus at the wrist 3 cm proximal to the distal wrist crease, at elbow near the volar crease of brachial pulse, and at axilla, recorded from abductor pollicis brevis with the reference electrode placed 3 cm distal at first metacarpophalangeal joint), ulnar nerves (supramaximal stimulus at wrist, ulnar groove, and axilla, recorded from abductor digiti minimi), common peroneal nerve (stimulation at ankle, 2 cm distal to the fibular neck and 5–8 cm above the fibular neck, surface reading recorded from extensor digitorum brevis), and posterior tibial nerve (stimulation behind and proximal to medial malleolus and popliteal fossa, surface reading recorded from abductor digiti quinti slight below and anterior to navicular bone). Sensory nerve conduction studies (SNCS) were done in median nerve (recorded from first interphalangeal joints of second digit and stimulations at wrist, elbow, and axilla), ulnar nerve (recorded from interphalangeal joints of fifth digit and stimulations at wrist, elbow, and axilla), and sural nerve (recorded between lateral malleolus and tendoachilles and stimulation 10–18 cm proximal to the recording electrode distal to the lower border of gastrocnemius at the junction of middle and lower third of leg). F-wave latency was recorded from all the motor nerves in the same setting.

The 100 normal values for each nerve variable (motor distal latencies, sensory latency, CMAP (compound muscle action potential), SNAP (sensory nerve action potential), motor CV (conduction velocity), sensory CV, and F-wave latency) were obtained by recording the neurophysiological values of 50 healthy individuals of similar age and height. This data was used to calculate the cut-off values to determine the peripheral nerve function (normal or abnormal) of 50 nondiabetic overweight and obese individuals. Volunteers were also classified on the basis of a number of nerves involved, namely, “<2” or “≥2.” Those volunteers whose nerve function was found to be normal (no nerves involved) and those with abnormal nerve function with only one nerve involved were categorized as “<2 nerves involved,” while the rest of the volunteers (abnormal nerve function with more than one nerve involved) were categorized as “≥2 nerves involved.”

The study did not include individuals with clear signs of polyneuropathy in the NCS and individuals with any diseases or drugs known to affect PNS (peripheral nervous system) function.

### 2.1. Statistical Analysis

The descriptive statistics for the baseline data from the study population were presented according to the main outcomes (dichotomous): nerve function (motor, sensory, and mixed) and number of nerves involved (motor, sensory, and mixed). All the continuous variables were tested for normality (Shapiro-Wilk test) and their significance of association with the outcome measurements was tested using Independent *t*-test if variables passed normality test; otherwise, Mann-Whitney *U* test was done. The descriptive values were expressed as mean ± SD (SD, standard deviation) when the variable passed the test of normality; otherwise, median (IQR) (IQR, interquartile range) was used. Similarly, *χ* ^2^ test was performed for categorical data. Multivariable logistic regression analysis was carried out in an explorative manner to control the confounders as well as predict outcomes. The variables (predictors) from bivariate analysis were selected at *P* ≤ 0.25 and these variables along with clinically relevant variables (FBG, blood glucose at 2 h (2hPP), fasting insulin, insulin at 2 h, HOMA-IR, and quantitative insulin sensitivity check index (QUICKI)) were subjected to binary logistic regression (entry at 0.05 and removal at 0.1) using backward stepwise method [9]. The statistically significant value was set at *P* < 0.05 for all the analyses.

## 3. Results

### 3.1. Descriptive Statistics

Out of 50 volunteers, the majority had abnormal nerve function of all kinds of nerves: motor (54%), sensory (72%), and mixed (80%). Most of them (70%) had ≥2 mixed nerves' involvement. Sensory nerves were more affected than motor nerves (54% versus 28%) (Table 1).

#### 3.1.1. Nerve Function

There was statistically significant difference in the mean of BMI (*P* = 0.048), FBG (*P* = 0.018), and 2hPP (*P* = 0.034) in volunteers with normal and abnormal motor nerve function. Higher BMI (33.57 kg/m^2^ versus 31.33 kg/m^2^), higher FBG (100.17 mg/dL versus 92.37 mg/dL), and higher 2hPP (128.10 mg/dL versus 114.40 mg/dL) were seen in volunteers with abnormal motor nerve function as compared to normal function (Table 2). None of the variables were significantly associated with both sensory and total nerve function (Tables 3 and 4). Though not statistically significant, BMI and HOMA-IR were seen higher in both abnormal sensory nerve function (BMI: 33.14 mg/dL versus 31.01 mg/dL; HOMA-IR: 2.45 versus 2.34) and abnormal mixed nerve function (BMI: 32.99 mg/dL versus 30.75 mg/dL; HOMA-IR: 2.45 versus 2.34) (Tables 3 and 4). A similar result was observed in the association of motor nerve function and HOMA-IR (*P* = 0.087, 2.07 in normal versus 2.73 in abnormal) (Table 2).

#### 3.1.2. Number of the Nerves Involved

The number of motor nerves involved was significantly associated with HOMA-IR and QUICKI at *P* = 0.039 and *P* = 0.049, respectively. A higher value of HOMA-IR (2.98 versus 2.19) and a lower value of QUICKI (0.32 versus 0.34) were observed in volunteers with ≥2 nerves involved than those with lesser number of nerves involved. Similarly, the association between BMI and the number of mixed nerves involved was statistically significant at *P* = 0.047 and higher BMI was seen in volunteers with ≥2 nerves involved as compared to those with <2 nerves involved (33.28 kg/m^2^, 30.82 kg/m^2^) (Table 4). BMI and HOMA-IR were higher when ≥2 nerves of all categories (except that HOMA-IR was slightly lesser in case of sensory nerve involvement) were involved as compared to <2 nerves involved; however, only the association of HOMA-IR with the number of motor nerves involved (Table 2) and the association of BMI with the number of mixed nerves involved (Table 4) were significant (*P* = 0.039 and *P* = 0.047, resp.).

### 3.2. Logistic Regression

#### 3.2.1. Nerve Function

Multivariable logistic regression (Model 1, Table 5) revealed increased FBG as the significant (*P* = 0.039, 95% CI = 1.003–1.127) predictor of abnormal motor nerve function. The report showed that an increase in 1 mg/dL FBG increased the odds of having an abnormal motor nerve function by 1.063 times. BMI showed a similar trend, but it was not significant. In case of sensory nerve function (Model 3, Table 7), HOMA-IR was the only significant predictor (*P* = 0.019, 96% CI = 1.420–49.322). It revealed that the value of HOMA-IR ≤2 increased the odds of abnormal sensory nerve function by 8.368 times compared to >2 HOMA-IR values. Though an increase in age and serum insulin level at 2 h also increased the odds of abnormal sensory nerve function by 1.084 and 1.014 times, respectively, the prediction was not statistically significant. Although weight gain, an increase in BMI, and decrease in HOMA-IR increased the odds of abnormal mixed nerve function, there were no significant predictors of mixed nerve function (Model 5, Table 9).

#### 3.2.2. Number of the Nerves Involved

Increase in age by one year (*P* = 0.015, 95% CI = 1.022–1.228) and serum insulin level at 2 h by one unit (*P* = 0.034, 95% CI = 1.001–1.020) significantly increased the odds of ≥2 motor nerves' involvement by 1.120 and 1.011 times, respectively (Model 2, Table 6). There were several statistically significant predictors of the number of sensory nerves' involvement (Model 4, Table 8). One-unit increase in BMI, FBG, fasting serum insulin, and serum insulin at 2 h increased the odds of ≥2 sensory nerves involved by 1.324, 1.327, 9.053, and 1.020 times, respectively, whereas a decrease in waist-hip ratio and HOMA-IR by one unit decreased the odds by <0.001 times. In case of mixed nerves (≥2 nerves) involvement, BMI was the only statistically significant predictor (Model 6, Table 10). An increase in BMI by 1 kg/m^2^ increased the odds of ≥2 mixed nerves involved by 1.267 times (*P* = 0.034, 95% CI = 1.018–1.577). Although one-unit decrease in HOMA-IR increased the odds of ≥2 mixed nerves involved and males have higher odds of mixed nerve involvement than females, both HOMA-IR and gender were not statistically significant.

## 4. Discussion

The influence of obesity on NCS parameters has not been studied adequately to date [10]. Most of the volunteers in our study had an abnormal nerve function of all kinds of nerves (motor, sensory, and mixed). Various parameters of NCS are affected by BMI [11]. Also, subclinical peripheral nerve impairment in obesity has been established [12] but to our knowledge the literature to date lacks any data quantitating peripheral nerves' involvement in overweight and obese volunteers as we evinced.

Majority had involvement of sensory nerves as compared to motor nerves. In previous studies, BMI was also found to have a negative correlation with sensory nerve action potential amplitude implying sensory axonal neuropathy [6, 12–15]. Thicker subcutaneous tissue has been denounced for sensory nerve amplitude involvement; however, this is purely hypothetical. In fact, McHugh et al. did not find BMI as a factor influencing nerve excitability [16]. Near nerve needle recording techniques may help resolve this issue [12].

Higher BMI, FBG, and 2hPP were observed in volunteers with abnormal motor nerve function. In a study by Pal et al., motor nerve latencies, decrease in the amplitude of action potentials, and conduction velocity were impaired in obesity [17]. The relation between obesity and impaired glycemic control is well known [18]. Motor nerve conduction is affected by obesity by creating a variety of comorbid conditions such as insulin resistance, diabetes, hypertension, hyperlipidemia, and hyperandrogenism [19]. The number of motor nerves involved was significantly associated with HOMA-IR and QUICKI. This finding is in contrast to the previous study done by Isojärvi et al., where high insulin level was observed to be beneficial for the function of the PNS [20]. This difference could be possible because of the difference in the number and types of nerves studied. We included routine upper limb and lower limb nerves whereas they had studied peroneal motor nerve conduction and radial, sural, and medial plantar sensory nerve conduction only. Also, we should remember that the actions of insulin on different types of nerves are yet to be determined.

Interestingly, in logistic regression, we found that increased FBG is the significant (*P* = 0.039, 95% CI = 1.003–1.127) predictor of abnormal motor nerve function. An increase in 1 mg/dL FBG increased the odds of having an abnormal motor nerve function by 1.063 times. Also, for obese volunteers to have ≥2 motor nerves' involvement, we found that age and serum insulin level at 2 h significantly increased the odds by 1.120 and 1.011 times, respectively. Singleton et al. in their study in patients with impaired glucose tolerance found 21% of them to have motor nerve involvement [5]. Although the protective role of insulin in PNS is described [21, 22], study done by Plastino et al. had shown detrimental effect [23]. The decrease in nerve conduction velocity and sensory amplitude associated with increasing age has been well documented [24, 25] and attributed to a decreased number of nerve fibers, reduction in fiber diameter, and changes in the fiber membrane [26]. Although the magnitude of change is relatively small within a narrow age range, it does affect predicted normal values [27]. Although meager, the literature suggests evidence of increasing motor dysfunction as age advances [28].

HOMA-IR was the only significant predictor of sensory nerve dysfunction. The value of HOMA-IR ≤2 increased the odds of abnormal sensory nerve function by 8.368 times compared to >2 HOMA-IR values, implying that insulin has a protective role which has been demonstrated by bountiful studies [20–22]. This protective role may be attributed to the fact that peripheral nerve expresses predominantly the high-affinity insulin receptor [29, 30].

BMI was the only significant predictor in case of mixed nerves' (≥2 nerves) involvement in our study. Peripheral nerve dysfunction is established in obesity [5, 6]. However, the mechanisms underlying it are still a matter of debate. Current evidence suggests the role of metabolic syndrome in the causation of neuropathy in obese patients [19]. However, the subcutaneous fat may also be responsible for altered nerve conduction parameters because obesity alone is not known to cause neuropathic changes pathologically [12]. So, whether the deranged parameters are pathological or just a normal finding in obese population is still unknown. Hence, some recommend separate NCS normative values for obese individuals [11] while others take these findings as a future risk for neuropathy [31].

We did not measure skin temperature, which is ideal during NCS, which is one of our study's major limitations. However, in routine practice, this may not be feasible.

## 5. Conclusion

Our study found evidence of deranged peripheral nerve function in overweight and obese population in various combinations. BMI, HOMA-IR, and FBG were found to be the significant predictors. Appropriate management strategies to control BMI, FBG, and insulin resistance could prevent adult overweight and obese individuals from a future neuropathic process.

## Figures and Tables

**Table 1:** Descriptive of outcome variables with nerve function and number of nerves involved in abnormality.

Type of nerves	Outcome variables			
	Peripheral nerve function (*N* = 50)		Number of nerves involved (*N* = 50)	
	Normal, *n* (%)	Abnormal, *n* (%)	<2, *n* (%)	≥2, *n* (%)
Motor nerve	23 (46)	27 (54)	36 (72)	14 (28)
Sensory nerve	14 (28)	36 (72)	23 (46)	27 (54)
Mixed nerve	10 (20)	40 (80)	15 (30)	35 (70)

**Table 2:** Baseline characteristics of volunteers according to the motor nerve. The values in the column of the motor nerves outside the small bracket are the count per total and those inside the bracket are the percentage.

	Motor nerves					
Variables	Peripheral nerve function			Number of nerves involved		
	Normal (*N* = 23)	Abnormal (*N* = 27)	*P* value	<2 (*N* = 36)	≥2 (*N* = 14)	*P* value
^‡^Age (years)	40.0 (13.0)	41.0 (13.0)	0.626	39.0 (13.0)	42.0 (22.0)	0.109
Gender (female)	21 (42)	24 (48)	1.000	31 (62)	14 (28)	0.304
Socioeconomic status			0.233			0.454
Lower	1 (2)	2 (4)		1 (2)	2 (4)	
Lower middle	3 (6)	10 (20)		9 (18)	4 (8)	
Upper lower	14 (28)	11 (22)		19 (38)	6 (12)	
Upper middle	5 (10)	4 (8)		7 (14)	2 (4)	
Alcohol consumer	2 (4)	2 (4)	1.000	4 (8)	0	0.566
Smoker	2 (4)	2 (4)	1.000	3 (6)	1 (2)	1.000
Exercise	6 (12)	8 (16)	0.781	10 (20)	4 (8)	1.000
Obesity in family	2 (4)	3 (6)	1.000	4 (8)	1 (2)	1.000
Diabetes in family	0	1 (2)	1.000	1 (2)	0	1.000
Hypertension history			0.235			0.013^*∗*^
None	16 (69.6)	18 (66.7)		27 (75)	7 (50.0)	
<1 to 5 years	7 (30.4)	6 (22.2)		9 (25)	4 (28.6)	
5 to >10 years	0	3 (11.1)		0	3 (21.4)	
Time of weight gained			0.275			0.008^*∗*^
<1 to 5 years	15 (65.2)	16 (59.3)		23 (63.9)	8 (57.1)	
5 to 10 years	4 (17.4)	3 (11.1)		6 (16.7)	1 (7.1)	
10 to 15 years	4 (17.4)	4 (14.8)		7 (19.4)	1 (7.1)	
15 to >20 years	0	4 (14.8)		0	4 (28.6)	
^‡^Height (m)	1.52 (0.06)	1.50 (0.08)	0.384	1.51 (0.06)	1.50 (0.10)	0.356
^†^Weight (kg)	72.78 ± 9.23	76.33 ± 10.62	0.217	74.67 ± 10.34	74.79 ± 9.70	0.971
^†^BMI (kg/m^2^)	31.33 ± 3.66	33.57 ± 4.10	0.048^*∗*^	32.29 ± 4.04	33.19 ± 4.05	0.485
BMI class			0.188			1.000
Obese-I	8 (34.8)	4 (14.8)		9 (25.0)	3 (21.4)	
Obese-II	15 (65.2)	23 (85.2)		27 (75.0)	11 (78.6)	
^†^Waist-hip ratio	0.86 ± 0.07	0.88 ± 0.07	0.268	0.87 ± 0.08	0.89 ± 0.05	0.305
^†^Waist-height ratio	0.61 ± 0.06	0.64 ± 0.06	0.050	0.62 ± 0.05	0.65 ± 0.06	0.086
^†^Fasting BGL (mg/dL)	92.37 ± 9.75	100.17 ± 12.29	0.018^*∗*^	95.77 ± 12.63	98.66 ± 9.23	0.442
^‡^BGL at 2 h (mg/dL)	114.4 (25.9)	128.1 (39.5)	0.034^*∗*^	121.9 (33.5)	127.85 (39.75)	0.437
^‡^Fasting serum insulin	9.96 (4.08)	11.18 (7.96)	0.227	9.98 (5.00)	12.37 (9.60)	0.054
^‡^Serum insulin at 2 h	62.2 (43.46)	64.41 (111.59)	0.381	62.68 (68.49)	70.51 (106.23)	0.130
hsCRP	6 (26.1)	9 (33.3)	0.804	10 (27.8)	5 (35.7)	0.733
^‡^HOMA-IR	2.07 (1.27)	2.73 (1.87)	0.087	2.19 (1.27)	2.98 (2.37)	0.039^*∗*^
HOMA-IR class			0.189			0.096
<2	11 (47.8)	7 (25.9)		16 (44.4)	2 (14.3)	
>2	12 (52.2)	20 (74.1)		20 (55.6)	12 (85.7)	
^†^QUICKI	0.34 ± 0.03	0.33 ± 0.02	0.133	0.34 ± 0.02	0.32 ± 0.02	0.049^*∗*^

BGL, blood glucose level; hsCRP, high sensitivity C-reactive protein.

^†^Mean ± SD, ^‡^median (IQR), and ^**∗**^significant at *P* < 0.05.

**Table 3:** Baseline characteristics of volunteers according to the sensory nerve. The values in the column of the sensory nerves outside the small bracket are the count per total and those inside the bracket are the percentage.

	Sensory nerves					
Variables	Peripheral nerve function			Number of nerves involved		
	Normal (*N* = 23)	Abnormal (*N* = 27)	*P* value	<2 (*N* = 36)	≥2 (*N* = 14)	*P* value
^‡^Age (years)	39.5 (15.0)	40.5 (12.0)	0.468	42.0 (14.0)	40.0 (12.0)	0.689
Gender (female)	12 (85.7)	33 (91.7)	0.611	20 (87.0)	25 (92.6)	0.651
Socioeconomic status			0.223			0.381
Lower	1 (7.1)	2 (5.6)		2 (8.7)	1 (3.7)	
Lower middle	3 (21.4)	10 (27.8)		6 (26.1)	7 (25.9)	
Upper lower	5 (35.7)	20 (55.6)		9 (39.1)	16 (59.3)	
Upper middle	5 (35.7)	4 (11.1)		6 (26.1)	3 (11.1)	
Alcohol consumer	1 (7.1)	3 (8.3)	1.000	2 (8.7)	2 (7.4)	1.000
Smoker	2 (14.3)	2 (5.6)	0.310	3 (13.0)	1 (3.7)	0.322
Exercise	4 (28.6)	10 (27.8)	1.000	5 (21.7)	9 (33.3)	0.552
Obesity in family	1 (7.1)	4 (11.1)	1.000	1 (4.3)	4 (14.8)	0.357
Diabetes in family	0	1 (2.8)	1.000	0	1 (3.7)	1.000
Hypertension history			0.535			0.754
None	10 (71.4)	24 (66.7)		15 (65.2)	19 (70.4)	
<1 to 5 years	4 (28.6)	9 (25.0)		7 (30.4)	6 (22.2)	
5 to >10 years	0	3 (8.3)		1 (4.3)	2 (7.4)	
Time of weight gained			0.741			0.949
<1 to 5 years	10 (71.4)	21 (58.3)		15 (65.2)	16 (59.3)	
5 to 10 years	2 (14.3)	5 (13.9)		3 (13.0)	4 (14.8)	
10 to 15 years	1 (7.1)	7 (19.4)		3 (13.0)	5 (18.5)	
15 to >20 years	1 (7.1)	3 (8.3)		2 (8.7)	2 (7.4)	
^‡^Height (m)	1.51 (0.04)	1.50 (0.09)	0.737	1.51 (0.05)	1.50 (0.09)	0.395
^†^Weight (kg)	70.93 ± 7.42	76.17 ± 10.66	0.099	73.22 ± 9.01	75.96 ± 10.90	0.342
^†^BMI (kg/m^2^)	31.01 ± 3.20	33.14 ± 4.19	0.093	31.41 ± 3.31	33.50 ± 4.37	0.067
BMI class			0.278			1.000
Obese-I	5 (35.7)	7 (19.4)		6 (26.1)	6 (22.2)	
Obese-II	9 (64.3)	29 (80.6)		17 (73.9)	21 (77.8)	
^†^Waist-hip ratio	0.89 ± 0.09	0.87 ± 0.06	0.305	0.89 ± 0.08	0.86 ± 0.07	0.231
^†^Waist-height ratio	0.63 ± 0.06	0.63 ± 0.06	0.843	0.63 ± 0.06	0.63 ± 0.06	0.680
^†^Fasting BGL (mg/dL)	95.45 ± 8.81	97.02 ± 12.81	0.676	97.19 ± 10.25	96.06 ± 13.07	0.740
^‡^BGL at 2 h (mg/dL)	116.3 (28.15)	124.5 (41.92)	0.280	114.7 (30.8)	125 (39.5)	0.192
^‡^Fasting serum insulin	10.27 (5.13)	10.19 (7.69)	0.846	10.10 (7.69)	10.34 (5.73)	0.755
^‡^Serum insulin at 2 h	60.77 (33.04)	65.30 (86.27)	0.531	60.83 (29.57)	76.60 (86.85)	0.471
hsCRP	3 (21.4)	12 (33.3)	0.507	5 (21.7)	10 (37.0)	0.386
^‡^HOMA-IR	2.34 (0.95)	2.45 (1.68)	0.923	2.45 (1.93)	2.43 (1.50)	0.668
HOMA-IR class			0.312			0.645
<2	3 (21.4)	15 (41.7)		7 (30.4)	11 (40.7)	
>2	11 (78.6)	21 (58.3)		16 (69.6)	16 (59.3)	
^†^QUICKI	0.33 ± 0.22	0.34 ± 0.03	0.77	0.33 ± 0.03	0.34 ± 0.03	0.651

^†^Mean ± SD, ^‡^median (IQR), and ^**∗**^significant at *P* < 0.05.

**Table 4:** Baseline characteristics of volunteers according to the total nerve. The values in the column of the mixed nerves outside the small bracket are the count per total and those inside the bracket are the percentage.

	Mixed nerves					
Variables	Peripheral nerve function			Number of nerves involved		
	Normal (*N* = 23)	Abnormal (*N* = 27)	*P* value	<2 (*N* = 36)	≥2 (*N* = 14)	*P* value
^‡^Age (years)	39.5 (13.0)	40.5 (13.0)	0.591	40.0 (14.0)	41.0 (13.0)	0.420
Gender (female)	9 (90)	36 (90)	1.000	12 (80)	33 (94.3)	0.152
Socioeconomic status			0.464			0.740
Lower	1 (10)	2 (5)		1 (6.7)	2 (5.7)	
Lower middle	1 (10)	12 (30)		3 (20)	10 (28.6)	
Upper lower	5 (50)	20 (50)		7 (46.7)	18 (51.4)	
Upper middle	3 (30)	6 (15)		4 (26.7)	5 (14.3)	
Alcohol consumer	1 (10.0)	3 (7.5)	1.000	2 (13.3)	2 (5.7)	0.574
Smoker	1 (10)	397.5)	1.000	2 (13.3)	2 (5.7)	0.574
Exercise	3 (30)	11 (27.5)	1.000	3 (20)	11 (31.4)	0.574
Obesity in family	1 (10)	4 (10)	1.000	1 (6.7)	4 (11.4)	1.000
Diabetes in family	0	1 (2.5)	1.000	0	1 (2.9)	1.000
Hypertension history			0.405			0.420
None	6 (60.0)	28 (70.0)		10 (66.7)	24 (68.6)	
<1 to 5 years	4 (40.0)	9 (22.5)		5 (33.3)	8 (22.9)	
5 to >10 years	0	3 (7.5)		0	3 (8.6)	
Time of weight gained			0.203			0.520
<1 to 5 years	9 (90)	22 (55)		11 (73.3)	20 (57.1)	
5 to 10 years	0	7 (17.5)		2 (13.3)	5 (14.3)	
10 to 15 years	1 (10)	7 (17.5)		2 (13.3)	6 (17.1)	
15 to >20 years	0	4 (10)		0	4 (11.4)	
^‡^Height (m)	1.52 (0.04)	1.50 (0.08)	0.558	1.52 (0.04)	1.50 (0.08)	0.457
^†^Weight (kg)	71.50 ± 7.59	75.50 ± 10.53	0.265	71.53 ± 6.61	76.06 ± 11.04	0.147
^†^BMI (kg/m^2^)	30.75 ± 3.17	32.99 ± 4.12	0.116	30.82 ± 2.85	33.28 ± 4.26	0.047^*∗*^
BMI class			0.225			0.471
Obese-I	4 (40)	8 (20)		5 (33.3)	7 (20.0)	
Obese-II	6 (60)	32 (80)		10 (66.7)	28 (80)	
^†^Waist-hip ratio	0.86 ± 0.08	0.88 ± 0.07	0.433	0.88 ± 0.09	0.87 ± 0.06	0.736
^†^Waist-height ratio	0.62 ± 0.06	0.63 ± 0.06	0.60	0.62 ± 0.06	0.63 ± 0.06	0.384
^†^Fasting BGL (mg/dL)	93.35 ± 7.24	97.39 ± 12.58	0.337	95.65 ± 11.24	96.98 ± 12.11	0.719
^‡^BGL at 2 h (mg/dL)	115.85 (30.83)	123.10 (41.92)	0.234	114.70 (31.90)	124.00 (30.90)	0.330
^‡^Fasting serum insulin	10.27 (5.78)	10.19 (6.72)	0.896	9.96 (3.85)	10.54 (7.83)	0.433
^‡^Serum insulin at 2 h	63.18 (33.04)	63.79 (83.19)	0.693	60.83 (35.73)	64.41 (84.52)	0.357
hsCRP	2 (20)	13 (32.5)	0.702	3 (20)	12 (34.3)	0.502
^‡^HOMA-IR	2.34 (1.42)	2.45 (1.66)	0.858	2.22 (1.05)	2.47 (1.78)	0.427
HOMA-IR class			0.730			1.000
<2	3 (30)	15 (37.5)		5 (33.3)	13 (37.1)	
>2	7 (70)	25 (62.5)		10 (66.7)	22 (62.9)	
^†^QUICKI	0.33 ± 0.03	0.34 ± 0.03	0.869	0.34 ± 0.03	0.33 ± 0.02	0.441

^†^Mean ± SD, ^‡^median (IQR), and ^**∗**^significant at *P* < 0.05.

**Table 5:** Model for predicting abnormal motor nerve function (Model 1).

Variables	*β* (SE)	*P* value	OR (95% CI)
BMI (kg/m^2^)	0.136 (0.081)	0.092	1.146 (0.978–1.342)
Fasting blood glucose (mg/dL)	0.061 (0.030)	0.039^*∗*^	1.063 (1.003–1.127)
Constant	−10.111	0.008	

Model *χ* ^2^ = 0.011, 59.980 (−2LL), 0.165 (Cox & Snell *R* ^2^), and 0.220 (Nagelkerke *R* ^2^); Hosmer and Lemeshow test: *P* = 0.211; SE: standard error.

^*∗*^significant at *P* < 0.05.

**Table 6:** Model for predicting ≥2 motor nerves involved (Model 2).

Variables	*β* (SE)	*P* value	OR (95% CI)
Age (years)	0.114 (0.047)	0.015^*∗*^	1.120 (1.022–1.228)
Serum insulin at 2 h (unit)	0.011 (0.005)	0.034^*∗*^	1.011 (1.001–1.020)
Constant	−6.720 (2.253)	0.003	

Model *χ* ^2^ = 0.006, 49.100 (−2LL), 0.184 (Cox & Snell *R* ^2^), and 0.266 (Nagelkerke *R* ^2^); Hosmer and Lemeshow test: *P* = 0.804.

^*∗*^significant at *P* < 0.05.

**Table 7:** Model for predicting abnormal sensory nerve function (Model 3).

Variables	*β* (SE)	*P* value	OR (95% CI)
Age (years)	0.080 (0.048)	0.093	1.084 (0.987–1.190)
≤2 HOMA-IR	2.124 (0.905)	0.019^*∗*^	8.368 (1.420–49.322)
Serum insulin at 2 h (unit)	0.014 (0.007)	0.052	1.014 (1.000–1.028)
Constant	−4.099 (2.300)	0.075	

Model *χ* ^2^ = 0.038, 50.877 (−2LL), 0.155 (Cox & Snell *R* ^2^), and 0.223 (Nagelkerke *R* ^2^); Hosmer and Lemeshow test: *P* = 0.153.

^*∗*^significant at *P* < 0.05.

**Table 8:** Model for predicting ≥2 sensory nerves involved (Model 4).

Variables	*β* (SE)	*P* value	OR (95% CI)
BMI (kg/m^2^)	0.280 (0.127)	0.028^*∗*^	1.324 (1.031–1.699)
Waist-hip ratio	−12.604 (6.396)	0.049^*∗*^	<0.001 (<0.001–0.934)
Fasting blood glucose (mg/dL)	0.283 (0.129)	0.028^*∗*^	1.327 (1.030–1.710)
Serum insulin at 0 h (unit)	2.203 (0.986)	0.026^*∗*^	9.053 (1.310–62.577)
Serum insulin at 2 h (unit)	0.019 (0.009)	0.025^*∗*^	1.020 (1.002–1.037)
HOMA-IR	−10.125 (4.360)	0.020^*∗*^	<0.001 (<0.001–0.206)
Constant	−24.125 (11.197)	0.031	

Model *χ* ^2^ = 0.002, 48.691 (−2LL), 0.334 (Cox & Snell *R* ^2^), and 0.446 (Nagelkerke *R* ^2^); Hosmer and Lemeshow test: *P* = 0.907.

^*∗*^significant at *P* < 0.05.

**Table 9:** Model for predicting abnormal mixed nerve function (Model 5).

Variables	*β* (SE)	*P* value	OR (95% CI)
Time of weight gained (years)	1.045 (0.648)	0.107	2.843 (0.799–10.119)
BMI (kg/m^2^)	0.245 (0.135)	0.070	1.277 (0.981–1.664)
HOMA-IR	−0.242 (0.216)	0.262	0.785 (0.514–1.198)
Constant	−7.188(4.126)	0.082	

Model *χ* ^2^ = 0.045, 42.013 (−2LL), 0.148 (Cox & Snell *R* ^2^), and 0.235 (Nagelkerke *R* ^2^); Hosmer and Lemeshow test: *P* = 0.803.

**Table 10:** Model for predicting ≥2 mixed nerves involved (Model 6).

Variables	*β* (SE)	*P* value	OR (95% CI)
Gender (male)	2.088 (1.189)	0.079	8.072 (0.786–82.917)
BMI (kg/m^2^)	0.237 (0.112)	0.034^*∗*^	1.267 (1.018–1.577)
HOMA-IR	−0.127 (0.206)	0.538	0.881 (0.589–1.318)
Constant	−10.317 (4.426)	0.020	

Model *χ* ^2^ = 0.042, 52.906 (−2LL), 0.151 (Cox & Snell *R* ^2^), and 0.214 (Nagelkerke *R* ^2^) and Hosmer and Lemeshow test: *P* = 0.702.

^*∗*^significant at *P* < 0.05.

## References

[1] Hughes R. A. C. (2002). Peripheral neuropathy. *The British Medical Journal*.

[2] Martyn C. N., Hughes R. A. C. (1997). Epidemiology of peripheral neuropathy. *Journal of Neurology, Neurosurgery & Psychiatry*.

[3] Boulton A. J. M. (2005). Management of diabetic peripheral neuropathy. *Clinical Diabetes*.

[4] Shanik M. H., Xu Y., Škrha J., Dankner R., Zick Y., Roth J. (2008). Insulin resistance and hyperinsulinemia: is hyperinsulinemia the cart or the horse?. *Diabetes Care*.

[5] Singleton J., Volckmann E., Graham T., Smith A. (2014). Neuropathy associated with nondiabetic obesity. *Neurology*.

[6] Miscio G., Guastamacchia G., Brunani A., Priano L., Baudo S., Mauro A. (2005). Obesity and peripheral neuropathy risk: a dangerous liaison. *Journal of the Peripheral Nervous System*.

[7] WHO The Asia-Pacific perspective: redefining obesity and its treatment. http://www.wpro.who.int/nutrition/documents/docs/Redefiningobesity.pdf.

[8] American Diabetes Association (ADA) (2008). Diagnosis and classification of diabetes mellitus. *Diabetes Care*.

[9] Steyerberg E. W., Eijkemans M. J. C., Harrell F. E., Habbema J. D. F. (2000). Prognostic modelling with logistic regression analysis: a comparison of selection and estimation methods in small data sets. *Statistics in Medicine*.

[10] Dorfman L. J., Robinson L. R. (1997). AAEM minimonograph #47: normative data in electrodiagnostic medicine. *Muscle & Nerve*.

[11] Pawar S. M., Taksande A. B., Singh R. (2012). Effect of body mass index on parameters of nerve conduction study in Indian population. *Indian Journal of Physiology and Pharmacology*.

[12] Buschbacher R. M. (1998). Body mass index effect on common nerve conduction study measurements. *Muscle and Nerve*.

[13] Hasanzadeh P., Oveisgharan S., Sedighi N., Nafissi S. (2008). Effect of skin thickness on sensory nerve action potential amplitude. *Clinical Neurophysiology*.

[14] Dorfman L. J., Robinson L. R. (1997). AAEM minimonograph #47: normative data in electrodiagnostic medicine. *Muscle Nerve*.

[15] Dumitru D., Dumitru D. (1995). Nerve conduction studies. *Electrodiagnostic Medicine*.

[16] McHugh J. C., Reilly R. B., Connolly S. (2011). Examining the effects of age, sex, and body mass index on normative median motor nerve excitability measurements. *Clinical Neurophysiology*.

[17] Pal P., Pal G., Balakumar B., Dutta T., Naik B. (2014). Assessment of motor nerve conduction in healthy obese Indian population. *International Journal of Clinical and Experimental Physiology*.

[18] Kahn S. E., Prigeon R. L., Mcculloch D. K. (1993). Quantification of the relationship between insulin sensitivity and *β*-cell function in human subjects: evidence for a hvoerbolic function. *Diabetes*.

[19] Flier J. S., Flier E. M., Braunwald E., Fauci A. S., Kasper D. L., Hauser S. L., Longo D. L., Jameson J. L. (2012). Nutrition: obesity. *Harrison's Principle of Internal Medicine*.

[20] Isojärvi H., Kallio M., Korpelainen R., Kaikkonen K., Jämsä T., Keinänen-Kiukaanniemi S. (2009). High insulin levels are positively associated with peripheral nervous system function. *Acta Neurologica Scandinavica*.

[21] Toth C., Brussee V., Martinez J. A., McDonald D., Cunningham F. A., Zochodne D. W. (2006). Rescue and regeneration of injured peripheral nerve axons by intrathecal insulin. *Neuroscience*.

[22] Ashraf A., Moghtaderi A. R., Yazdani A. H., Mirshams S. (2009). Evaluation of effectiveness of local insulin injection in none insulin dependent diabetic patient with carpal tunnel syndrome. *Electromyography and Clinical Neurophysiology*.

[23] Plastino M., Fava A., Carmela C. (2011). Insulin resistance increases risk of carpal tunnel syndrome: a case-control study. *Journal of the Peripheral Nervous System*.

[24] Buchthal F., Rosenfalck A. (1966). Evoked action potentials and conduction velocity in human sensory nerves. *Brain Research*.

[25] Kemble F. (1967). Conduction in the normal adult median nerve: the different effect of ageing in men and women. *Electromyography*.

[26] Mayer R. F. (1963). Nerve conduction studies in man. *Neurology*.

[27] Stetson D. S., Albers J. W., Silverstein B. A., Wolfe R. A. (1992). Effects of age, sex, and anthropometric factors on nerve conduction measures. *Muscle and Nerve*.

[28] Metter E. J., Conwit R., Metter B., Pacheco T., Tobin J. (1998). The relationship of peripheral motor nerve conduction velocity to age- associated loss of grip strength. *Aging Clinical and Experimental Research*.

[29] Sugimoto K., Murakawa Y., Zhang W., Xu G., Sima A. A. F. (2000). Insulin receptor in rat peripheral nerve: its localization and alternatively spliced isoforms. *Diabetes/Metabolism Research and Reviews*.

[30] Waldbillig R. J., LeRoith D. (1987). Insulin receptors in the peripheral nervous system: a structural and functional analysis. *Brain Research*.

[31] Yadav R. L., Khadka R., Thakur D., Agrawal K., Paude1 B. Nerve conduction study and heart rate variability in obese persons.

